# Universal Transcutaneous Bilirubin Screening in a Midwifery-Led Home Care Setting

**DOI:** 10.1001/jamanetworkopen.2025.51883

**Published:** 2026-01-12

**Authors:** Lauren E. H. Westenberg, Marten J. Poley, Helene A. Bouma, Daan Nieboer, Berthe A. M. van der Geest, Andrei Tintu, Jolande Y. Vis, Henk Groen, Erwin Ista, Peter H. Dijk, Eric A. P. Steegers, Irwin K. M. Reiss, Christian V. Hulzebos, Jasper V. Been

**Affiliations:** 1 Division of Neonatology, Department of Neonatal and Pediatric Intensive Care, Erasmus MC Sophia Children’s Hospital, University Medical Centre Rotterdam, Rotterdam, the Netherlands; 2 Department of Obstetrics and Gynecology, Erasmus MC Sophia Children’s Hospital, University Medical Centre Rotterdam, Rotterdam, the Netherlands; 3 Erasmus School of Health Policy & Management and institute for Medical Technology Assessment (iMTA), Erasmus University Rotterdam, Rotterdam, the Netherlands; 4 Department of Pediatric Surgery, Sophia Children’s Hospital, Erasmus MC, Rotterdam, the Netherlands; 5 Division of Neonatology, Department of Pediatrics, Beatrix Children’s Hospital, University Medical Centre Groningen, Groningen, the Netherlands; 6 Department of Public Health, Erasmus MC, University Medical Centre Rotterdam, Rotterdam, the Netherlands; 7 Department of Clinical Chemistry, Erasmus MC, University Medical Centre Rotterdam, Rotterdam, the Netherlands; 8 Department of Epidemiology, University of Groningen, University Medical Centre Groningen, Groningen, the Netherlands; 9 Nursing Science, Department of Internal Medicine, Erasmus MC, University Medical Centre Rotterdam, Rotterdam, the Netherlands; 10 Division of Pediatric Intensive Care, Department Neonatal and Pediatric Intensive Care, Erasmus MC Sophia Children’s Hospital, University Medical Centre Rotterdam, Rotterdam, the Netherlands; 11 University Hospital Eppendorf, Division of Neonatology and Pediatric Intensive Care, Children University Hospital Eppendorf, Hamburg, Germany

## Abstract

**Importance:**

Visual inspection is commonly the first-line screening method for hyperbilirubinemia in neonates cared for at home. However, it is unreliable and may delay treatment.

**Objective:**

To assess the clinical outcomes, costs, and user convenience associated with universal transcutaneous bilirubin (TCB) screening for detecting hyperbilirubinemia in a diverse neonatal population cared for at home.

**Design, Setting, and Participants:**

This prospective decision analytical model was conducted between July 11, 2021, and June 9, 2023, in 9 midwifery practices across the Netherlands. Neonates (gestational age ≥35 weeks) were eligible if they had their first midwife visit prior to postnatal day 6 and had not yet received phototherapy.

**Exposure:**

At each home visit, the midwife first undertook visual inspection and then performed TCB measurements on the neonate’s sternum. Bilirubin was measured in blood when indicated by visual inspection and/or elevated TCB reading.

**Main Outcomes and Measures:**

The hypothesis was that TCB could replace visual inspection if (1) more neonates requiring treatment for hyperbilirubinemia were detected and (2) fewer heel pricks were needed. The 2 main outcomes were (1) blood bilirubin level greater than the national guideline’s treatment threshold and (2) requiring a heel prick for bilirubin quantification. McNemar tests were used for both hypotheses. Cost-effectiveness was assessed using a decision-tree analytic model.

**Results:**

Data from 2314 neonates (median [IQR] gestational age, 39 [39-40] weeks; 1172 [50.6%] male) were analyzed. Overall, 78 (3.4%) had a bilirubin level greater than the treatment threshold. Of these, 28 had been identified through TCB screening but missed by visual inspection (absolute risk difference, 28%; 95% CI, 13%-42%; *P* < .001). Although TCB missed 7 neonates identified through visual inspection, these were all attributable to either misinterpretation of TCB readings by the midwife or structural overestimation of blood bilirubin levels in one hospital laboratory. TCB screening led to 102 additional heel pricks vs visual inspection (244 vs 142; *P* < .001). Sensitivity analysis showed that when TCB screening was selectively applied only to neonates with any jaundice, additional heel pricks were reduced to 82 (*P* < .001), with no loss in diagnostic performance. Universal TCB screening saved €15 ($17) per neonate.

**Conclusions and Relevance:**

In this decision analytical model study of 2314 (near) full-term neonates, universal TCB screening among neonates cared for at home identified more neonates requiring treatment but required more heel pricks than visual inspection alone. Universal TCB screening was cost-effective compared with visual inspection. The additional required heel pricks decreased by selective TCB screening.

## Key Points

**Question:**

Does universal transcutaneous bilirubin (TCB) screening identify more neonates who require phototherapy compared with visual inspection, and is this strategy cost-effective?

**Findings:**

In this decision analytical model study of 2314 (near) full-term neonates cared for at home, universal TCB screening identified 21 additional cases of hyperbilirubinemia compared with visual inspection, saving €15 per neonate but requiring 102 extra heel pricks. Selective TCB screening for neonates with visual jaundice reduced additional heel pricks without compromising diagnostic accuracy.

**Meaning:**

These findings support the use of TCB screening for neonates at home.

## Teaser

This decision analytical model compares the outcomes and costs associated with universal transcutaneous bilirubin screening vs visual inspection for identifying neonates being cared for at home in the Netherlands who require phototherapy.

## Introduction

Neonatal hyperbilirubinemia results from a metabolic imbalance in bilirubin production, conjugation, and clearance.^1^ Hyperbilirubinemia is typically mild and self-limiting, but in some cases, it can progress to severe neonatal hyperbilirubinemia (SNH), which in turn may lead to bilirubin-induced neurotoxic effects.^2^ Although entirely preventable, the global burden of SNH and the consequential lifelong morbidity and even mortality are still substantial.^3,4^ Neonatal jaundice care remains challenging, particularly screening and identifying neonates at risk of hyperbilirubinemia necessitating treatment.^5,6,7,8,9^

Hospital-based screening programs using noninvasive transcutaneous bilirubin (TCB) quantification have been shown to effectively prevent SNH.^10,11,12^ As such, TCB assessment is recommended before hospital discharge in multiple international guidelines.^13,14^ However, globally, home-based neonatal care is common, with many (near) full-term neonates receiving care at home during the first postnatal week.^15,16,17^ To our knowledge, no large-scale prospective study has evaluated the outcomes and costs associated with TCB screening in neonates cared for at home. At home, jaundice screening still commonly relies on visual inspection, although its inaccuracy has been long recognized.^18,19^ A recent national perinatal audit program showed continued occurrence of SNH in the home setting.^8^ Universal TCB screening may be a cost-effective approach, given its potential to prevent the adverse health and economic consequences of SNH. At the same time, TCB meters are expensive and require costly annual calibration. Therefore, robust evidence is required to confirm whether this strategy truly provides good value for money.

We undertook a prospective multicenter decision analytical model study to assess the costs and outcomes of universal TCB screening for detecting hyperbilirubinemia necessitating treatment in a diverse neonatal population cared for at home. The 2 co–primary outcomes were number of neonates with hyperbilirubinemia necessitating treatment and number of heel pricks taken to quantify total serum bilirubin (TSB) which had a level less than the treatment threshold.

## Methods

### Study Design

The Better Assessment of Neonatal Jaundice at Home (BEAT jaundice @home) study was a prospective multicenter decision analytical model study conducted in 9 Dutch community midwifery practices between July 11, 2021, and June 9, 2023. The study received ethical approval from the Erasmus MC Medical Research Ethics Committee and was registered at the Netherlands Trial Register (NL9545). Parents or caregivers gave written informed consent. Reporting complies with the Standards for Reporting of Diagnostic Accuracy Studies (STARD) reporting guideline.^20^ Detailed study methods were published previously.^21^ A text box explaining the Dutch birth care system in which the study was conducted can be found in eAppendix 1 in Supplement 1.

### Participants

Neonates were eligible if they were born at a gestational age of 35 weeks or later, were at home at any point between days 2 and 8 of life, and had their first midwife visit at home before postnatal day 6. Neonates were ineligible if they had previously received phototherapy or had parents or caregivers who had insufficient understanding of the Dutch language.

### Test Methods

At each midwife visit, visual inspection of jaundice was performed first. Only after this result had been recorded, the TCB device (Draeger JM-105 [Draegerwerk]) was applied at the neonate’s sternum. TCB measurements were plotted on a customized version of the Dutch bilirubin nomogram, which included a safety margin of 2.92 mg/dL (50 µmol/L) (eAppendix 2 in Supplement 1). As such, 3 TCB-based nomograms were constructed that were 2.92 mg/dL (50 µmol/L) lower than the corresponding TSB-based nomograms. The decision to quantify TSB was made either when the midwife felt this was clinically indicated based on visual jaundice or when TCB was at or greater than the TCB threshold. TCB devices used in this study were calibrated yearly by the manufacturer. Community midwives were trained by the study team in the use and interpretation of TCB screening.

### Intended Sample Size

A prespecified sample size calculation was undertaken to address the 2 primary hypotheses based on the TSB level. This was based on hypothetical screening findings at each midwife visit categorized according to visual inspection and TCB levels, as informed by published literature (eAppendix 3 in Supplement 1).^22^

For the first hypothesis, which tests whether universal TCB screening would detect more neonates requiring treatment than visual inspection, the required sample size for 80% power at an α of 5% was 2100 neonates, which would produce an expected 38 neonates requiring treatment.^23^ For the second hypothesis, which tests whether universal TCB screening would reduce the number of unnecessary heel pricks (ie, heel pricks with a TSB level less than the threshold) vs visual inspection,^24^ 260 neonates were required to achieve 80% power at an α of 5%. Based on the required sample size for the first hypothesis and anticipating a 10% loss to follow-up, we aimed to include 2310 neonates.

### Statistical Analysis

Descriptive analyses were used to summarize clinical data. The primary objective was to evaluate whether universal TCB screening (index test) compared with visual inspection (reference test) would increase the detection of hyperbilirubinemia necessitating treatment (target condition), while reducing the need for heel pricks to quantify bilirubin levels. For the first hypothesis, a McNemar test was used conditional on having a TSB level greater than the treatment threshold according to the reading in the laboratory. For the second hypothesis, an adjusted McNemar test was used conditional on having a TSB level less than the threshold, taking into account the repeated measurements. Secondary analyses included 2 × 2 tables to calculate positive predictive values, negative predictive values, sensitivity, and specificity. A Bland-Altman plot was used to calculate the mean difference and corresponding 95% limits of agreement between TCB and TSB. A prespecified subgroup analysis was performed according to gestational age strata (ie, less than 38 weeks of gestation or 38 weeks of gestation or greater).^25^ We had also prespecified a subgroup analysis by ethnicity, which we did by skin tone as classified by the Fitzpatrick skin scale by parents or caregivers.^26^

Additionally, we undertook several post hoc analyses: (1) a sensitivity analysis to assess performance of selective TCB screening (ie, applied only to neonates with any degree of visual jaundice), (2) a per-protocol analysis (ie, according to whether blood for TSB should have been taken rather than whether it actually was taken, thus accounting for occasional misinterpretation of TCB values by midwives in relation to the nomograms), and (3) a sensitivity analysis excluding neonates whose TSB level was quantified in a specific hospital laboratory out of the 13 participating laboratories. This was because it became apparent during the study that TSB levels were systematically overestimated in this laboratory. SPSS version 28.0.1.0 (IBM Corp) and R studio version 4.3.2 (R Project for Statistical Computing) were used for the statistical analyses. Statistical significance was set at *P* < .05.

#### Cost-Effectiveness Analysis 

A decision-tree model was built, as screening for neonatal hyperbilirubinemia can be represented by a relatively simple sequence of decisions.^27^ The analysis was performed from a health care perspective, using the study’s 14 days’ time horizon. As such, discounting of future costs and effects was not needed. The analysis adhered to recommended methodologies for economic evaluations in health care.^28,29,30^ Reporting of the cost-effectiveness analysis (CEA) followed the Consolidated Health Economic Evaluation Reporting Standards (CHEERS) reporting guidelines.^31^

The model simulated a hypothetical neonate receiving either visual inspection or TCB screening. Probabilities used in the model were based on the current study. Assumptions were made for neonates who required treatment but were initially missed by visual inspection,^8,32,33^ which was necessary to estimate their outcomes in the absence of TCB screening within the decision-tree model. All relevant health care resource use was considered in the model inputs. To determine costs, resource use was multiplied by integral cost prices based on Dutch reference prices and on data received from equipment manufacturers,^34^ calculated in Euros and expressed in 2025 price levels.

The measures used in the CEA were (1) the number of correctly diagnosed cases of hyperbilirubinemia necessitating treatment and (2) the number of heel pricks avoided. Costs and outcomes were determined for each branch of the decision tree. A strategy was considered dominant if it was both more effective and less costly. If dominance was not achieved, incremental cost-effectiveness ratios (ICERs) were calculated. ICERs represent the additional cost required to gain 1 extra unit of health effect when adopting a new treatment strategy.^35^ One-way sensitivity analyses were carried out to assess robustness of the results to plausible variations in underlying key parameters and assumptions.

#### User Convenience

The community midwives were invited for interviews focused on how they integrated the TCB into their daily practices, any challenges they encountered, and their overall impressions of its effectiveness. Participation was voluntary, and interviews were organized during and after the study. Interviews were semistructured and were conducted both individually and in group settings.

## Results

**Figure 1.**
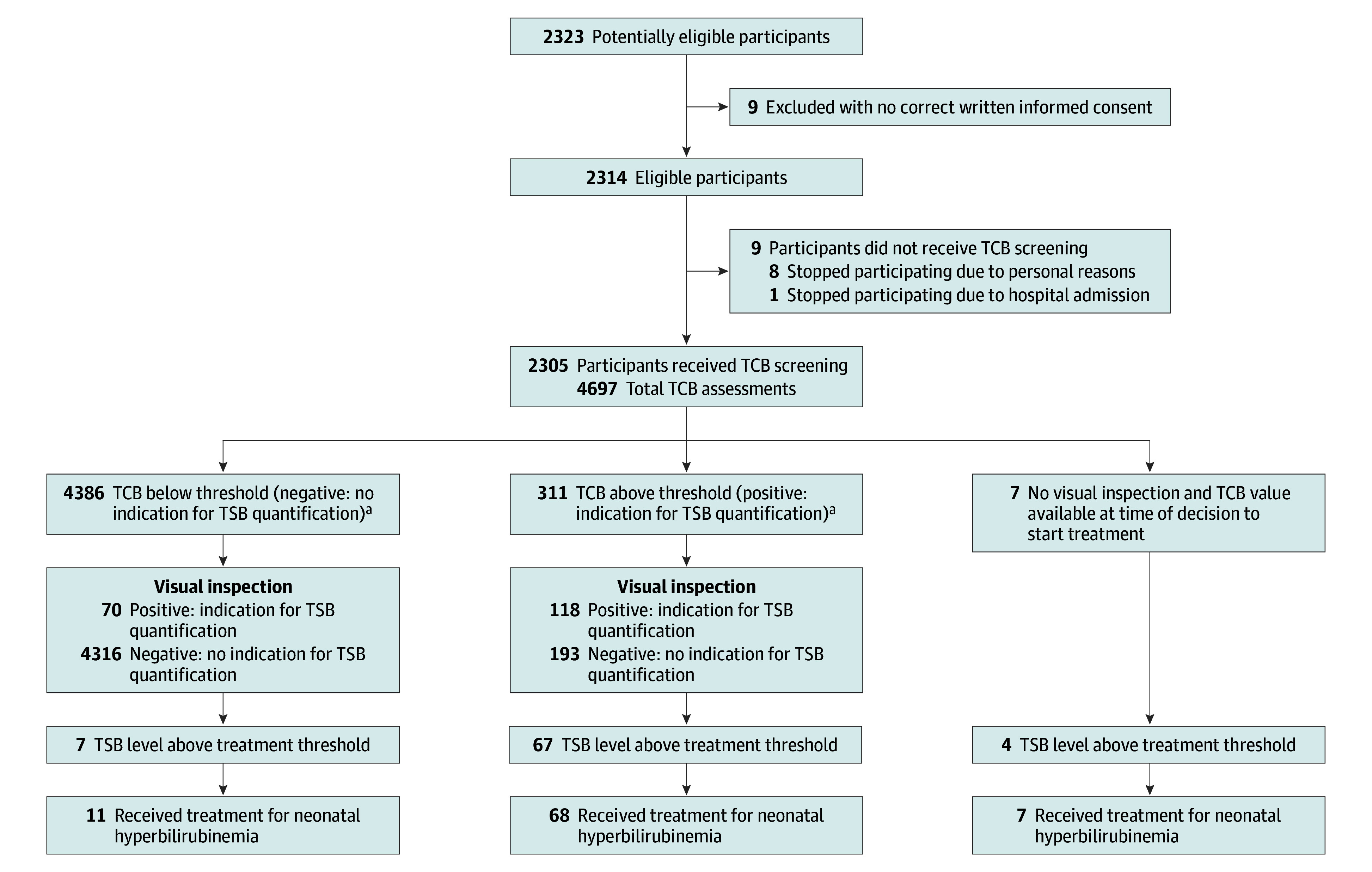
Flow Diagram of Participants During the Study TCB indicates transcutaneous bilirubin; TSB, total serum bilirubin. ^a^According to the interpretation of the community midwives.

Of the 2323 neonates recruited, 2314 (median [IQR] gestational age, 39 [39-40] weeks; 1172 [50.6%] male) had valid written informed consent, and 2305 participants had complete follow-up data. The community midwives made a median (IQR) of 2 (1-3) home visits. In total, 4697 paired assessments of visual inspection and TCB screening were conducted, and 423 blood samples were taken and analyzed in 13 laboratories. Figure 1 displays the flow diagram of participants throughout the study. The Table displays the clinical characteristics of the participants.

**Table.** Demographic and Clinical Characteristics of the Participants

Characteristic	Participants, No. (%) (N = 2314)
**Delivery characteristics**	
Gravidity, median (IQR)	2 (1-3)
Parity, median (IQR)	1 (0-1)
Type of delivery	
Vaginal delivery	1883 (81.4)
Instrumental vaginal delivery	169 (7.3)
Cesarean delivery	262 (11.3)
Cephalohematoma	
Yes	5 (0.2)
No	2309 (99.8)
Apgar score <5 at 5 min	
Yes	19 (0.8)
No	2295 (99.2)
Umbilical cord blood pH <7.0	
Yes	9 (0.4)
No	809 (35.0)
Not determined	1496 (64.6)
**Characteristics of the biological parent(s)**	
Maternal blood group	
O	1044 (45.1)
A	917 (39.6)
B	245 (10.6)
AB	77 (3.3)
Unknown	31 (1.3)
Maternal rhesus D type	
Rhesus D positive	1950 (84.3)
Rhesus D negative	339 (14.6)
Unknown	25 (1.1)
Maternal Fitzpatrick Skin scale^a^	
I	408 (17.5)
II	1368 (59.1)
III	289 (12.5)
IV	144 (6.2)
V	79 (3.4)
VI	30 (1.3)
Paternal Fitzpatrick Skin scale^a^	
I	326 (14.1)
II	1327 (57.3)
III	316 (13.7)
IV	162 (7.0)
V	118 (5.1)
VI	48 (2.1)
Unknown	17 (0.7)
**Characteristics of the neonate**	
Gestational age, wk	
Median (IQR)	39 (39-40)
<38	180 (7.8)
≥38	2134 (92.2)
Sex	
Male	1172 (50.6)
Female	1142 (49.4)
Birth weight, median (IQR), g	3515 (3204-3787)
Type of feeding in first 2 weeks of life	
Breastfeeding	1299 (56.1)
Formula	419 (18.1)
Mixed	590 (25.5)
Missing	6 (0.3)
Neonatal blood group	
O	10 (0.4)
A	10 (0.4)
B	8 (0.3)
AB	1 (0.1)
Unknown or not determined	2285 (98.8)
Fetal or neonatal rhesus D factor	
Rhesus D positive	224 (9.6)
Rhesus D negative	131 (5.7)
Not determined	1962 (84.8)
DAT test	
Positive	3 (0.1)
Negative	26 (1.1)
Unknown or not determined	2285 (98.8)
Loss of birth weight >10% in first week of life	
Yes	35 (1.5)
No	2273 (98.2)
Unknown	6 (0.3)
Large for gestational age due to maternal diabetes	
Yes	1 (0.1)
No	2313 (99.9)
Risk factors for hyperbilirubinemia and neurotoxicity according to the Dutch national guideline^15^	
ABO incompatibility	
Yes	12 (0.5)
No	17 (0.7)
Unknown	2285 (98.8)
Ill or drowsy baby	
Yes	9 (0.4)
No	2305 (99.6)
Glucose-6-phosphate dehydrogenase deficiency	
Yes	1 (0.1)
No or unknown	2313 (99.9)
Bilirubin nomogram risk category^b^	
Lower risk	2110 (91.2)
Medium risk	200 (8.6)
Higher risk	4 (0.2)
Other characteristics	
Siblings received treatment for neonatal hyperbilirubinemia	
Yes	48 (2.1)
No	2266 (97.9)
Characteristics of treatment for neonatal hyperbilirubinemia	
Exchange transfusion	0
Phototherapy	86 (3.7)
Received treatment in hospital	79 (92.0)
Received treatment at home	7 (8.0)
Median duration of phototherapy, d	
Received treatment in hospital, median (IQR)	1.0 (1.0-2.0)
Received treatment at home, median (IQR)	2.0 (1.75-2.25)
Received treatment twice	1 (1.2)

Abbreviation: DAT, direct antiglobulin test.

^a^ According to the Fitzpatrick skin scale as determined by parents or caregivers themselves.^26^

^b^ According to the community midwives.

Overall, 78 neonates (3.4%) had the primary outcome of a TSB level greater than the treatment threshold (Figure 1). Of these, 70 (89.7%) actually received treatment for neonatal hyperbilirubinemia. The remaining 8 participants (10.3%) were managed expectantly and subsequently had declining TSB levels. In total, 86 neonates (3.7%) were treated for hyperbilirubinemia. Sixteen participants had a TSB level just less than the treatment threshold but a clinical indication for treatment as determined by the consultant pediatrician, such as a steep rise in the TSB level. Although 4 neonates initially had TSB levels greater than the exchange transfusion threshold, they responded well to (intensive) phototherapy and no exchange transfusions were required. No complications of SNH were reported in any of the participants at follow-up.

### Primary Outcomes

**Figure 2.**
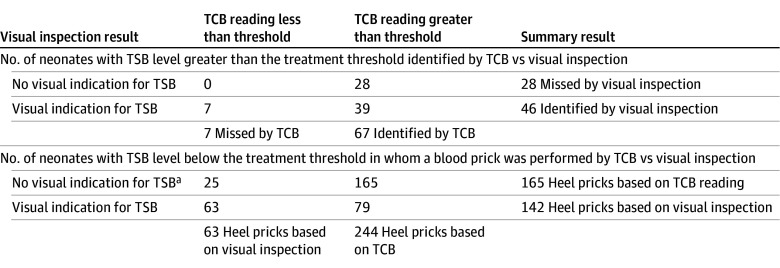
Primary Outcomes: TCB Screening vs Visual Inspection TCB indicates transcutaneous bilirubin; TSB, total serum bilirubin. ^a^Other reasons for heel prick included request by parents or caregivers or advise from maternity care assistant or consultant pediatrician.

Of the 78 neonates (3.4%) who had a TSB level above the treatment threshold, a concurrent TCB reading was available for 74 (Figure 2). Of these, 28 had been identified through TCB screening but were missed by visual inspection, while 7 were only identified through visual inspection alone. As such, TCB screening identified 21 more neonates requiring treatment than visual inspection did (absolute risk difference: 28%; 95% CI, 13%-42%; *P* < .001). Of note, 2 neonates missed by visual inspection had TSB levels above the exchange transfusion threshold. Based on TCB readings, 244 unnecessary heel pricks were performed, whereas 142 would have been performed if only visual inspection had been applied (*P* < .001) (Figure 2).

### Subgroup Analyses

Effectiveness of TCB screening did not vary by gestational age group or across different skin tones. Full results of subgroup analyses appear in eAppendix 4 and eAppendix 5 in Supplement 1.

### Secondary Outcomes

TCB had a 21.5% positive predictive value and 92.6% negative predictive value for having a TSB level above the treatment threshold (eAppendix 6 in Supplement 1). Secondary outcomes for visual inspection can be found in eAppendix 7 in Supplement 1. Based on 392 paired measurements of TCB and TSB, mean difference was 0.04 (95% limits of agreement, −6.81 to 6.88) mg/dL (0.7 [95% limits of agreement, −116.4 to 117.7] µmol/L) (eAppendix 8 in Supplement 1).

### Post Hoc Analyses

Selective TCB screening identified 20 additional neonates with a TSB greater than the treatment threshold vs visual inspection (eAppendix 9 in Supplement 1). Selective screening reduced the number of unnecessary heel pricks from 102 to 82 as compared with universal screening (eAppendix 9 in Supplement 1).

In the per-protocol analysis, TCB screening identified 26 additional neonates with a TSB greater than the treatment threshold vs visual inspection (*P* < .001). This led to 160 additional heel pricks (eAppendix 10 in Supplement 1).

During the study it became evident through the semistructured interviews with the participating community midwives that 1 of the 13 participating laboratories structurally overestimated TSB levels. In the sensitivity analysis excluding neonates assessed in this particular laboratory, TCB identified 7 additional neonates compared with visual inspection, which increased to 10 in a per-protocol analysis. In the latter analysis, no neonates were missed by TCB screening (eAppendix 11 in Supplement 1).

### Cost-Effectiveness

**Figure 3.**
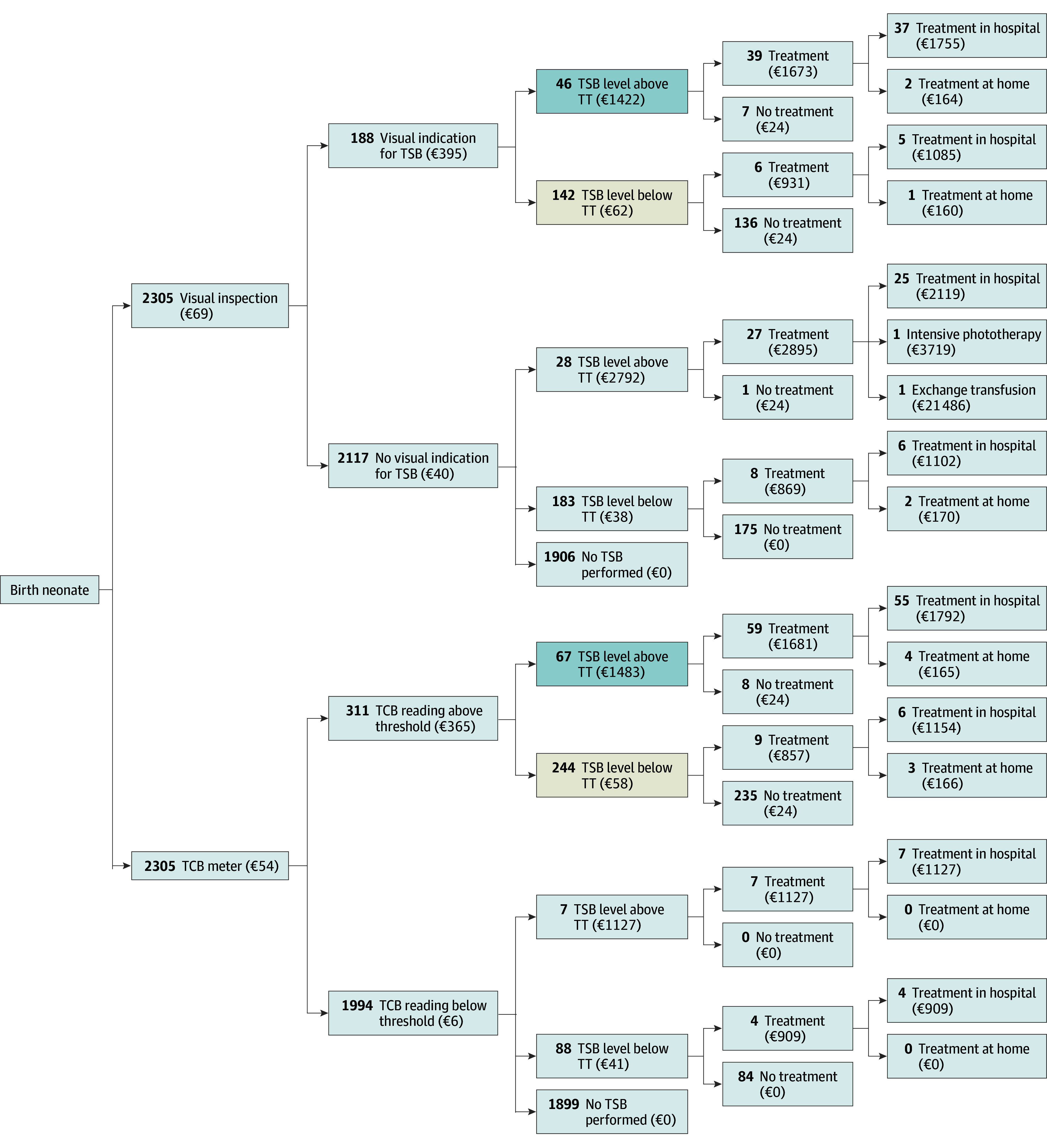
Decision Tree Analytic Model for Universal Transcutaneous Bilirubin (TCB) and Visual Inspection Screening for Neonatal Hyperbilirubinemia The decision tree presents, for each pathway, the number of neonates and the costs per neonate (to convert Euros to US dollars, multiply by 1.15). In terms of the outcome measures of the cost-effectiveness analysis, correctly diagnosed cases of hyperbilirubinemia necessitating treatment are dark gray, while the number of unnecessary heel pricks is tan. TSB indicates total serum bilirubin; TT, treatment threshold.

Over the 14-day time horizon, TCB correctly diagnosed 67 neonates with hyperbilirubinemia necessitating treatment, compared with 46 correctly diagnosed in the visual inspection scenario. However, TCB screening also resulted in 244 unnecessary heel pricks compared with 142 with visual inspection. Costs were calculated using the unit prices (eAppendix 12 in Supplement 1). Regarding the overall economic benefits, universal TCB screening incurred costs of €54 ($62) per neonate vs €69 ($80) for visual inspection, resulting in mean savings of €15 ($17) per neonate (Figure 3). As such, TCB screening had greater estimated effectiveness, by correctly diagnosing more neonates necessitating treatment, and less costly than visual inspection. Since dominance was not achieved when considering the incremental cost per heel prick avoided, an ICER was calculated for that outcome. TCB screening overall led to cost savings compared with visual inspection, but it was also associated with more unnecessary heel pricks. The ICER indicated that €374 ($431) was saved for every additional unnecessary heel prick.

**Figure 4.**
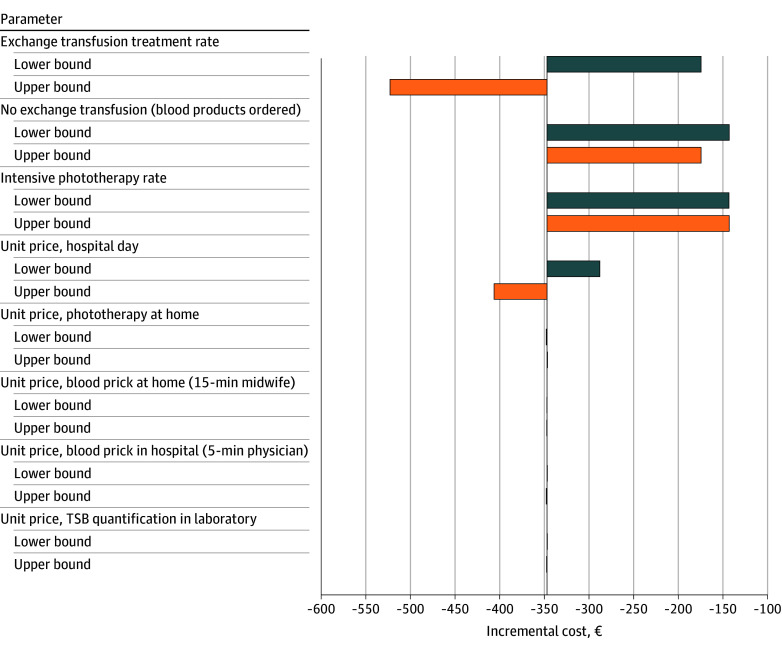
Tornado Diagram Displaying the Impact of Varying Parameters on Incremental Cost Per Heel Prick Avoided To convert Euros to US dollars, multiply by 1.15.

In 1-way sensitivity analyses, 4 uncertain parameters were varied: the number of neonates requiring exchange transfusion, the number of neonates for whom blood products were ordered in anticipation of a possible exchange transfusion, the numbers of neonates requiring intensive phototherapy, and multiple unit cost prices (eAppendix 12 in Supplement 1). The results were most sensitive to exchange transfusion treatments, blood products ordered, intensive phototherapy treatment rate, and the unit cost price of a hospital day (Figure 4). TCB screening was associated with less cost and better outcomes in terms of correctly diagnosing neonates necessitating treatment across all sensitivity analyses.

### User Convenience

The midwives generally expressed satisfaction with the TCB device. They highlighted several facilitators. First, the TCB device was easy to operate, and the midwives found it straightforward to explain its use to their direct colleagues. Second, the TCB device, being an objective instrument, helped reduce disagreements with other health care professionals. However, the midwives also noted that heel pricks to quantify bilirubin levels were more frequent. Barriers to implementation, as described by the midwives were (1) the costs of purchase and yearly calibration by the manufacturer and (2) lack of a uniform protocol for home care.

## Discussion

In this large prospective multicenter decision analytical model study among more than 2300 (near) full-term neonates, universal TCB screening identified 28 additional neonates requiring hyperbilirubinemia treatment who were missed by visual inspection. Two of these neonates had bilirubin levels that exceeded the threshold for exchange transfusion. Universal TCB screening led to 102 additional heel pricks. As such, 6 extra heel pricks were required to identify 1 additional neonate requiring treatment. While the primary analysis indicated that 7 neonates with visual jaundice and elevated TSB were missed by TCB screening, post hoc sensitivity analyses indicated that this was attributable to either misinterpretation of TCB readings by the midwives or structural TSB overestimation in one laboratory. Universal TCB screening saved €15 per neonate compared with visual inspection. Taken together, these findings suggest that universal TCB screening at home is associated with cost-effective improvements in the identification of neonates requiring treatment compared with visual inspection, although at the expense of some additional unnecessary heel pricks.

Our study differed from previous studies regarding TCB screening in various aspects. First, our study was conducted in the home setting, whereas previous research was mostly carried out in hospital environments, which does not easily translate to primary care or home settings.^36,37^ Although study protocols initiating TCB screening programs in primary care or home settings are available,^21,22,38^ clinical recommendations for TCB screening made in international guidelines are restricted to the predischarge period.^13,15^ This does not translate well to the situation in the Netherlands and other countries, where many neonates are either cared for at home from birth or discharged home shortly after institutional birth. A recent Cochrane review included 1 randomized clinical trial (RCT) evaluating the effectiveness of TCB screening compared with visual inspection for hyperbilirubinemia in a clinical setting. TCB screening reduced hospital readmissions for hyperbilirubinemia likely because it increased phototherapy treatment prior to discharge.^37^ Another recent Cochrane review^17^ included 23 studies determining the diagnostic accuracy of TCB screening for detecting neonatal hyperbilirubinemia. None of these studies evaluated the newest Draeger JM-105 device,^36^ which was used in our study.

Based on previous literature, such as a Dutch hospital-based RCT showing that selective TCB screening reduced the number of blood pricks by 38.5%, we hypothesized that our study would also show a decrease.^10,39^ The fact that we found the opposite likely indicates that in current practice the threshold for taking blood samples in the home setting based on visual inspection is too high. This probably explains the continued occurrence of SNH in the home setting in the Netherlands.^8^ Our findings indicate that this situation may be significantly improved by transitioning from visual inspection to TCB screening as a first-line screening tool.

This is the first study to our knowledge that used real patient data to assess the cost-effectiveness of universal TCB screening in the home setting.^40,41^ In the current era of rising health care costs, cost-effectiveness has become increasingly important.^35,42^ Findings from this study provide valuable evidence on both the clinical and economic value of TCB screening in neonates cared for at home. These results can help inform health care policymakers considering public funding of TCB screening as well as professional organizations evaluating its inclusion in clinical practice guidelines. The cost savings identified are likely attributable to earlier identification of neonates necessitating treatment through TCB screening. Earlier treatment could prevent the need for escalation to intensive or prolonged phototherapy or even exchange transfusion. The economic evaluation necessarily used a short time horizon. A longer time horizon could capture additional health benefits from preventing bilirubin-induced neurotoxic effects, which suggests that the cost-effectiveness of TCB screening may be even greater than our findings indicate. In our primary analysis, 7 neonates with TSB levels greater than the treatment threshold were missed by TCB screening. Post hoc analyses indicated that all of these cases could be explained by either misinterpretation of the TCB reading against the threshold or by TSB levels having been overestimated in one specific laboratory. These findings suggest that universal TCB screening may safely replace visual inspection in the home setting. A further post hoc analysis indicated that selective TCB screening has the potential to reduce the number of unnecessary blood pricks. Although this approach missed 1 neonate in our study who was not visually jaundiced, TSB levels were only marginally elevated in this case and likely overestimated due to bias of the TSB quantification in the aforementioned hospital laboratory. Taken together, our study findings suggest that selective TCB screening may be as safe as universal TCB screening in the home setting, while leading to fewer unnecessary blood pricks.

The BEAT study was undertaken in a unique home care setting, addressing key challenges of doing research in a setting where this is not yet commonplace. Nonetheless, we achieved the predetermined sample size for both primary outcomes within the planned time frame.^21^ The study design was especially distinctive due to the collaboration and coordination between health care workers in both the first and second tier of the perinatal care system. We are unaware of similar studies having been conducted at this scale in the primary care setting.

Findings from our study underscore the potential benefits of implementing TCB screening for (near) full-term neonates in home care. TCB screening was associated with improved early detection of neonates requiring treatment by identifying additional cases of hyperbilirubinemia that would have been missed with visual inspection alone. Hence, TCB screening can help reduce the incidence of SNH and its related complications. While TCB screening may lead to an increase in heel pricks, the overall benefit of accurately identifying at-risk neonates requiring treatment clearly outweighs this drawback. The fact that TCB screening picked up 2 neonates with TSB values greater than the exchange transfusion threshold, who were missed with visual inspection, emphasizes this point. We recommend integrating TCB screening into routine postnatal care in agreement with the recent revised Dutch guideline on management of hyperbilirubinemia.^15^ Considering the misinterpretation of TCB readings, which resulted in neonates incorrectly being missed by the screening, we recommend comprehensive education and training programs to ensure that midwives are proficient in using TCB devices and interpreting the readings accurately. We advocate developing standardized protocols for TCB screening tailored to home settings to ensure quality of care after hospital discharge. In addition, a quality control system to identify and correct discrepancies in TCB and TSB measurement, for example due to laboratory overestimations as observed in the current study, should be practiced.

### Limitations

This study has limitations. It was observational and therefore not blinded. However, the midwives were systematically trained to first record the findings from visual inspection and decide whether to draw blood based on visual inspection alone, before performing TCB measurements. This approach likely minimized potential interference or bias between the 2 screening methods. Another limitation is the use of the skin tone of the parents or caregivers as a reference, since there is no internationally recognized skin tone scale available for neonates.^43^ This could impact results of the subgroup analysis. Third, one hospital laboratory measured roughly one-third of the TSB samples. In hindsight, structural overestimation of TSB values by this laboratory led to false positives, which biased the primary analysis. Our sensitivity and per-protocol analyses showed that several false negatives of the TCB device were attributable to this overestimation. Following the BEAT study, this hospital laboratory adjusted its methods to quantify bilirubin. Other false negatives were due to misinterpretation of TCB readings against the thresholds by the midwives. Moreover, due to the partial verification of disease status there is a small potential for bias in sensitivity and specificity estimates. Finally, although severe neonatal hyperbilirubinemia is still occurring in the Netherlands,^8^ our study was conducted in a high-resource setting with an established clinical infrastructure. As such, generalizability to high-risk populations in middle- and low-resource settings is limited.

## Conclusions

In this unique large-scale prospective decision analytical model study, universal TCB screening for neonates cared for at home was cost-effective in identifying neonates needing treatment compared with visual inspection. The number of additional heel pricks associated with this approach can likely be reduced by selective screening at no loss of diagnostic performance. Our findings emphasize the need for improved and coordinated management of neonatal jaundice care.

## Notes

Supplement 1. **eAppendix 1.** The Dutch Birth Care System **eAppendix 2.** Customized Transcutaneous Bilirubin Nomogram **eAppendix 3.** Probability Matrix Distribution of Participants for Sample Size Calculation **eAppendix 4.** Corresponding 2 × 2 Tables Subgroup Analysis by Gestational Age Strata **eAppendix 5.** Corresponding 2 × 2 Tables Subgroup Analysis by Fitzpatrick Skin Scale **eAppendix 6.** Corresponding 2 × 2 Table for Positive Predictive Value, Negative Predictive Value, Sensitivity, and Specificity for TCB **eAppendix 7.** Corresponding 2 × 2 Table for Positive Predictive Value, Negative Predictive Value, Sensitivity, and Specificity for Visual Inspection **eAppendix 8.** Bland-Altman Plot of TCB Measurements vs TSB Measurements in µmol/L **eAppendix 9.** Corresponding 2 × 2 Tables Sensitivity Analysis for Selective Screening **eAppendix 10.** Corresponding 2 × 2 Tables for Per-Protocol Analysis **eAppendix 11.** Corresponding 2 × 2 Tables Sensitivity Analysis Excluded Neonates Assessed in a Particular Hospital-Laboratory **eAppendix 12.** Unit Prices Used in the Cost-Effectiveness Analysis

Supplement 2.
Nonauthor Collaborators

Supplement 3.
Data Sharing Statement
