# Supplementary material for: Universal Transcutaneous Bilirubin Screening in a Midwifery-Led Home Care Setting
Source: JAMA Netw Open. 2026 Jan 12;9(1):e2551883. doi: 10.1001/jamanetworkopen.2025.51883 (PMC12797098; doi:10.1001/jamanetworkopen.2025.51883)
Supplement: Supplement 1. — eAppendix 1. The Dutch Birth Care System eAppendix 2. Customized Transcutaneous Bilirubin Nomogram eAppendix 3. Probability Matrix Distribution of Participants for Sample Size Calculation eAppendix 4. Corresponding 2 × 2 Tables Subgroup Analysis by Gestational Age Strata eAppendix 5. Corresponding 2 × 2 Tables Subgroup Analysis by Fitzpatrick Skin Scale eAppendix 6. Corresponding 2 × 2 Table for Positive Predictive Value, Negative Predictive Value, Sensitivity, and Specificity for TCB eAppendix 7. Corresponding 2 × 2 Table for Positive Predictive Value, Negative Predictive Value, Sensitivity, and Specificity for Visual Inspection eAppendix 8. Bland-Altman Plot of TCB Measurements vs TSB Measurements in µmol/L eAppendix 9. Corresponding 2 × 2 Tables Sensitivity Analysis for Selective Screening eAppendix 10. Corresponding 2 × 2 Tables for Per-Protocol Analysis eAppendix 11. Corresponding 2 × 2 Tables Sensitivity Analysis Excluded Neonates Assessed in a Particular Hospital-Laboratory eAppendix 12. Unit Prices Used in the Cost-Effectiveness Analysis [file jamanetwopen-e2551883-s001.pdf]

Supplemental Online Content

Westenberg LH, Poley MJ, Bouma HA, et al; BEAT study group. Universal transcutaneous bilirubin screening in a midwifery-led home care setting. *JAMA Netw Open.* 2026;9(1):e2551883. doi:10.1001/jamanetworkopen.2025.51883

eAppendix 1. The Dutch Birth Care System

eAppendix 2. Customized Transcutaneous Bilirubin Nomogram

eAppendix 3. Probability Matrix Distribution of Participants for Sample Size Calculation

eAppendix 4. Corresponding 2 × 2 Tables Subgroup Analysis by Gestational Age Strata

eAppendix 5. Corresponding 2 × 2 Tables Subgroup Analysis by Fitzpatrick Skin Scale

eAppendix 6. Corresponding 2 × 2 Table for Positive Predictive Value, Negative Predictive Value, Sensitivity, and Specificity for TcB

eAppendix 7. Corresponding 2 × 2 Table for Positive Predictive Value, Negative Predictive Value, Sensitivity, and Specificity for Visual Inspection

eAppendix 8. Bland-Altman Plot of TcB Measurements vs TSB Measurements in µmol/L

eAppendix 9. Corresponding 2 × 2 Tables Sensitivity Analysis for Selective Screening

eAppendix 10. Corresponding 2 × 2 Tables for Per-Protocol Analysis

eAppendix 11. Corresponding 2 × 2 Tables Sensitivity Analysis Excluded Neonates Assessed in a Particular Hospital-Laboratory

eAppendix 12. Unit Prices Used in the Cost-Effectiveness Analysis

This supplemental material has been provided by the authors to give readers additional information about their work.

© 2026 Westenberg LH et al. *JAMA Network Open*.

eAppendix 1. The Dutch Birth Care System

TEXT BOX: THE DUTCH BIRTH CARE SYSTEM

In the Netherlands, the majority of neonates are cared for at home during the first postnatal week, regardless of the place of birth. Daily postpartum care is provided by maternity care assistants during the first week after birth, under the supervision of community midwives. A midwife visits the family two to three times during the first week. The registration of risk factors and visual inspection of the neonate's color of skin and sclerae is the standard screening method for neonates cared for at home, in accordance with the national guideline.(1) If potentially severe hyperbilirubinemia is suspected based on visual inspection, the community midwife may decide to check the TSB, via a blood sample taken by the midwife or by a specialized laboratory home service. The sample is then taken to the nearest hospital laboratory for TSB analysis. Based on the TSB, the need for treatment of hyperbilirubinemia is assessed using the treatment nomogram from the Dutch national guideline on hyperbilirubinemia, adapted from the American Academy of Pediatrics' 2004 guideline.(2) The nomogram includes three nomograms that indicate the need for phototherapy, based on the TSB level (in µmol/L), postnatal age (in hours), and a number of

© 2026 Westenberg LH et al. *JAMA Network Open*.

eAppendix 2. Customized Transcutaneous Bilirubin Nomogram

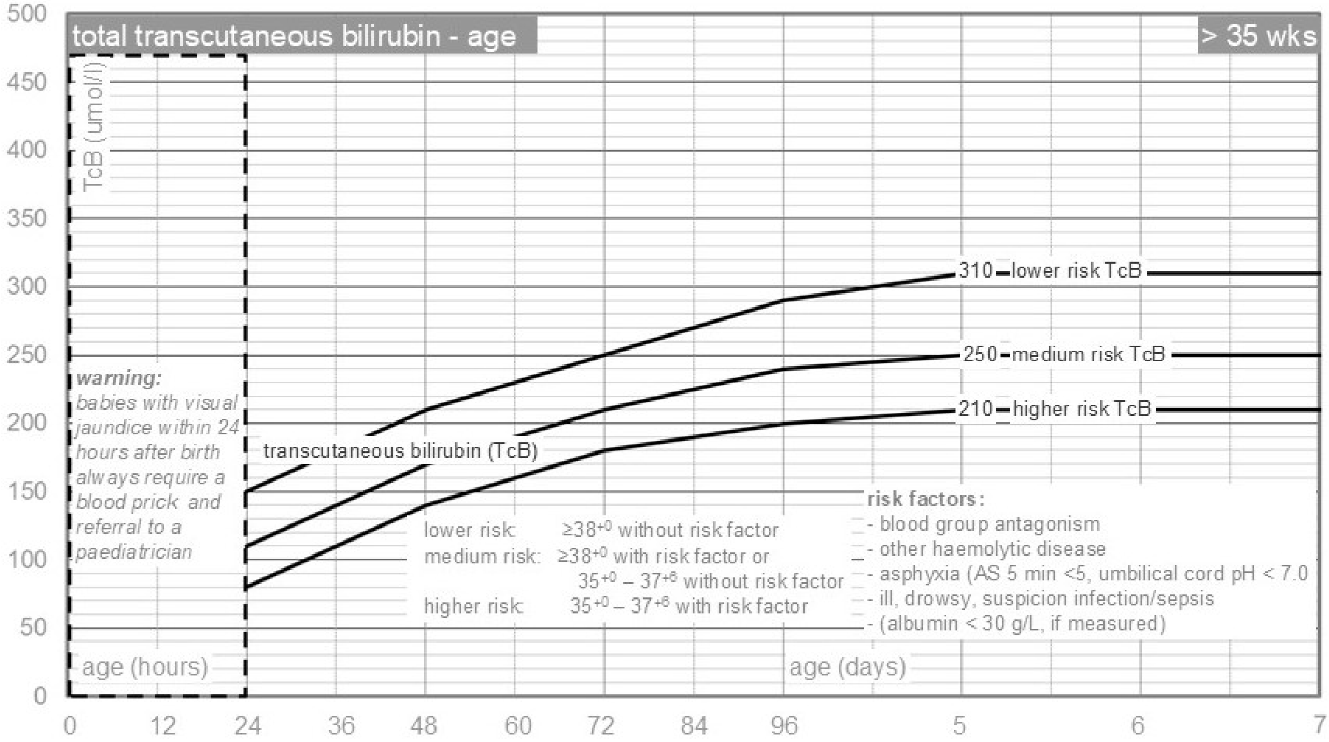

Abbreviations: TcB, transcutaneous bilirubin; TSB, AS, apgar score

© 2026 Westenberg LH et al. *JAMA Network Open*.

eAppendix 3. Probability Matrix Distribution of Participants for Sample Size Calculation

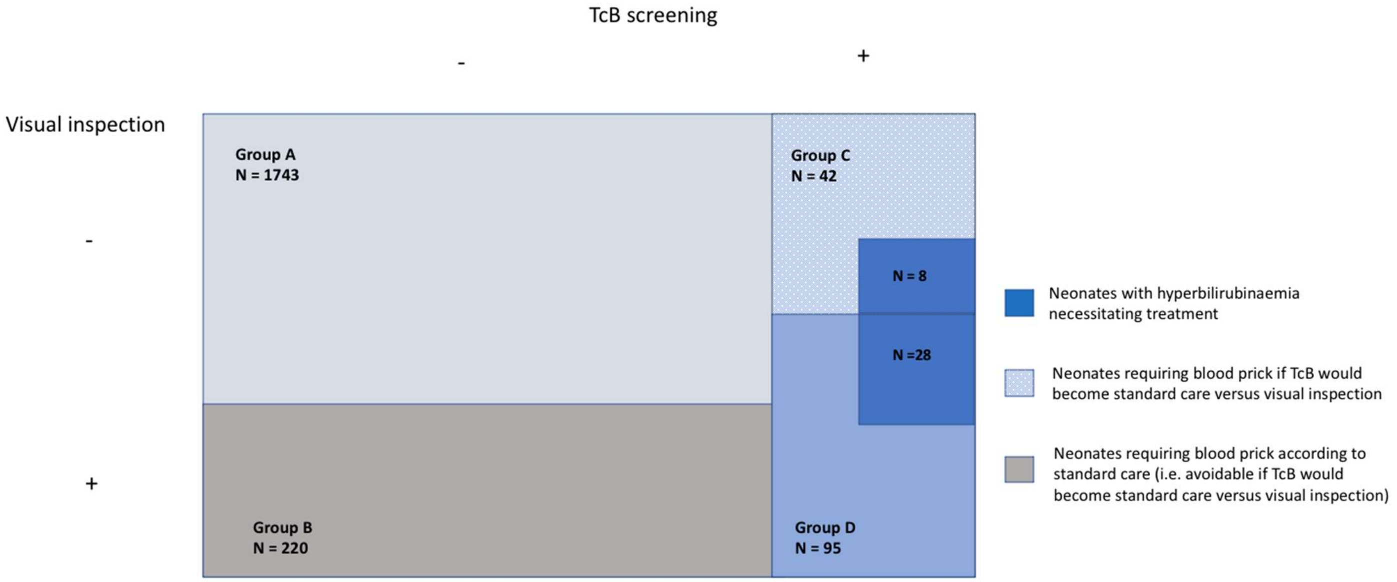

Abbreviations: *TcB=transcutaneous bilirubinometry*

Anticipated numbers of neonates falling in each of the four groups across the observations

© 2026 Westenberg LH et al. *JAMA Network Open*.

eAppendix 4. Corresponding 2 × 2 Tables Subgroup Analysis by Gestational Age Strata

4A – Subgroup: gestational age below 38 weeks

Number of neonates with TSB level above the treatment threshold identified by TcB versus visual inspection

	TcB reading below threshold	TcB reading above threshold	
No visual indication for TSB	0	12	Missed by visual inspection: 12
Visual indication for TSB	3	10	Identified by visual inspection: 13
	Missed by TcB: 3	Identified by TcB: 22	

Abbreviations: TcB, transcutaneous bilirubin; TSB, total serum bilirubin

Number of neonates with TSB level below the treatment threshold requiring unnecessary blood pricks by TcB versus visual inspection

	TcB reading below threshold	TcB reading above threshold
No visual indication for TSB*	3	50
Visual indication for TSB*	8	25

Abbreviations: TcB, transcutaneous bilirubin; TSB, total serum bilirubin

* Other reasons for heel prick included request by parent(s)/caregiver(s), or advise from maternity care assistant or consultant pediatrician

4B – Subgroup: gestational age 38 weeks or above

Number of neonates with TSB level above the treatment threshold identified by TcB versus visual inspection

	TcB reading below threshold	TcB reading above threshold	
No visual indication for TSB	0	16	Missed by visual inspection: 16
Visual indication for TSB	4	29	Identified by visual inspection: 33
	Missed by TcB: 4	Identified by TcB: 45	

Abbreviations: TcB, transcutaneous bilirubin; TSB, total serum bilirubin

Number of neonates with TSB level below the treatment threshold requiring unnecessary blood pricks by TcB versus visual inspection

	TcB reading below threshold	TcB reading above threshold
No visual indication for TSB*	22	115
Visual indication for TSB*	55	54

Abbreviations: TcB, transcutaneous bilirubin; TSB, total serum bilirubin

* Other reasons for heel prick included request by parent(s)/caregiver(s), or advise from maternity care assistant or consultant pediatrician

An interaction test showed no significant differences in the additional number of neonates requiring treatment between the different gestational age groups (P=1.000). For the neonates requiring a heel prick the interaction test showed significant differences between gestational age groups (P<0.001).

© 2026 Westenberg LH et al. *JAMA Network Open*.

eAppendix 5. Corresponding 2 × 2 Tables Subgroup Analysis by Fitzpatrick Skin Scale

5A – Subgroup: Fitzpatrick skin scale I-III group

Number of neonates with TSB level above the treatment threshold identified by TcB versus visual inspection

	TcB reading below threshold	TcB reading above threshold	
No visual indication for TSB	0	23	Missed by visual inspection: 23
Visual indication for TSB	6	31	Identified by visual inspection: 37
	Missed by TcB: 6	Identified by TcB: 44	

Abbreviations: TcB, transcutaneous bilirubin; TSB, total serum bilirubin

Number of neonates with TSB level below the treatment threshold requiring unnecessary blood pricks by TcB versus visual inspection

	TcB reading below threshold	TcB reading above threshold
No visual indication for TSB*	21	121
Visual indication for TSB*	52	56

Abbreviations: TcB, transcutaneous bilirubin; TSB, total serum bilirubin

* Other reasons for heel prick included request by parent(s)/caregiver(s), or advise from maternity care assistant or consultant pediatrician

5B – Subgroup: Fitzpatrick skin scale IV-VI group

Number of neonates with TSB level above the treatment threshold identified by TcB versus visual inspection

	TcB reading below threshold	TcB reading above threshold	
No visual indication for TSB	0	5	Missed by visual inspection: 5
Visual indication for TSB	1	8	Identified by visual inspection: 9
	Missed by TcB: 1	Identified by TcB: 13	

Abbreviations: TcB, transcutaneous bilirubin; TSB, total serum bilirubin

Number of neonates with TSB level below the treatment threshold requiring unnecessary blood pricks by TcB versus visual inspection

	TcB reading below threshold	TcB reading above threshold
No visual indication for TSB*	4	44
Visual indication for TSB*	11	23

Abbreviations: TcB, transcutaneous bilirubin; TSB, total serum bilirubin

* Other reasons for heel prick included request by parent(s)/caregiver(s), or advise from maternity care assistant or consultant pediatrician

An interaction test showed no significant differences in the additional number of neonates requiring treatment between the different gestational age groups (P=0.827). For the neonates requiring a heel prick the interaction test showed significant differences between gestational age groups (P<0.001).

© 2026 Westenberg LH et al. *JAMA Network Open*.

eAppendix 6. Corresponding 2 × 2 Table for Positive Predictive Value, Negative Predictive Value, Sensitivity, and Specificity for TcB

	TSB above threshold	TSB below threshold	
TcB reading above threshold*	TP=67	FP=244	PPV: 67/311=21.5%
TcB reading below threshold*	FN=7	TN=88	NPV: 88/95=92.6%
	Sensitivity: 67/74=90.5%	Specificity: 88/332=26.5%	

Abbreviations: TcB, transcutaneous bilirubin; TSB, total serum bilirubin; TP, true positive; FP, false positive; FN, false negative; TN, true negative; PPV, positive predictive value; NPV, negative predictive value
* According to the community midwives

© 2026 Westenberg LH et al. *JAMA Network Open*.

eAppendix 7. Corresponding 2 × 2 Table for Positive Predictive Value, Negative Predictive Value, Sensitivity, and Specificity for Visual Inspection

	TSB above threshold	TSB below threshold	
**Positive visual inspection**	TP=46	FP=142	PPV: 46/188 = 23.6%
**Negative visual inspection**	FN=28	TN=183	NPV: 183/211 = 86.7%
	Sensitivity: 46/74 = 62.2%	Specificity: 183/325 = 56.2%	

Abbreviations: TSB, total serum bilirubin

© 2026 Westenberg LH et al. *JAMA Network Open*.

eAppendix 8. Bland-Altman Plot of TcB Measurements vs TSB Measurements in µmol/L

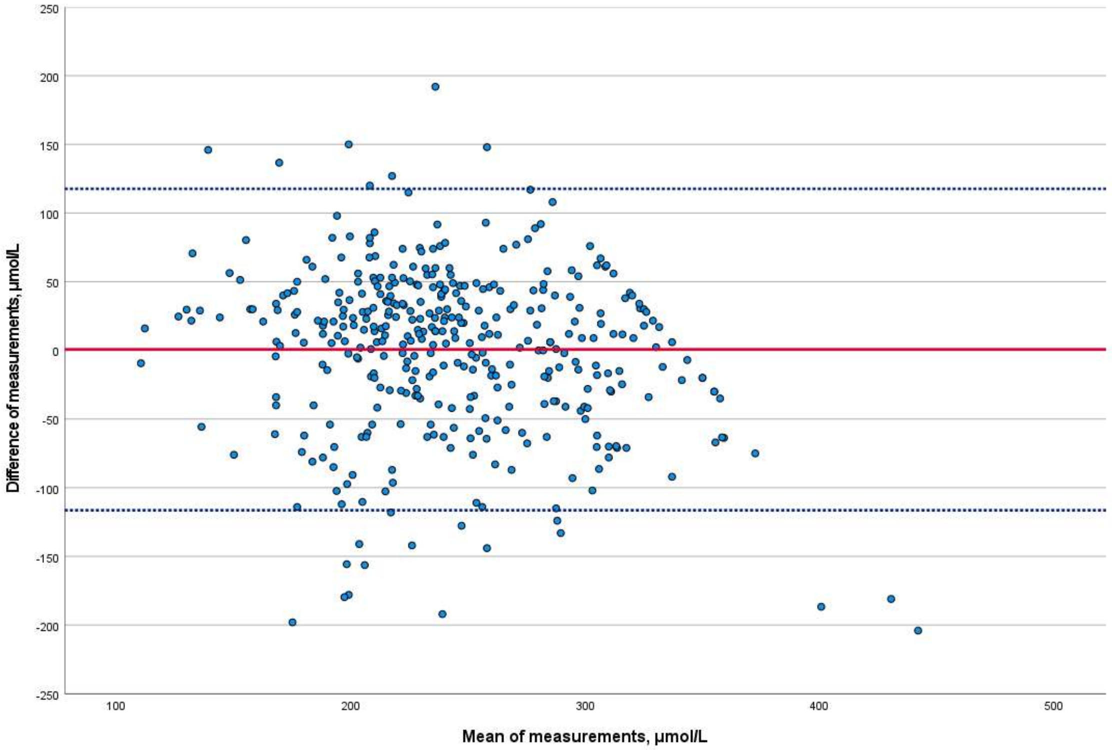

Abbreviations: TcB, transcutaneous bilirubin; TSB, total serum bilirubin

© 2026 Westenberg LH et al. *JAMA Network Open*.

eAppendix 9. Corresponding 2 × 2 Tables Sensitivity Analysis for Selective Screening

Number of neonates with TSB level above the treatment threshold identified by TcB versus visual inspection

	TcB reading below threshold	TcB reading above threshold	
No visual indication for TSB	0	27	Missed by visual inspection: 20
Visual indication for TSB	7	39	Identified by visual inspection: 46
	Missed by TcB: 7	Identified by TcB: 66	

Abbreviations: TcB, transcutaneous bilirubin; TSB, total serum bilirubin

Number of neonates with TSB level below the treatment threshold requiring unnecessary blood pricks by TcB versus visual inspection

	TcB reading below threshold	TcB reading above threshold
No visual indication for TSB*	23	145
Visual indication for TSB*	63	79

Abbreviations: TcB, transcutaneous bilirubin; TSB, total serum bilirubin
* Other reasons for heel prick included request by parent(s)/caregiver(s), or advise from maternity care assistant or consultant pediatrician

© 2026 Westenberg LH et al. *JAMA Network Open*.

eAppendix 10. Corresponding 2 × 2 Tables for Per-Protocol Analysis

Number of neonates with TSB level above the treatment threshold identified by TcB versus visual inspection

	TcB reading below threshold	TcB reading above threshold	
**No visual indication for TSB**	0	28	Missed by visual inspection: 28
**Visual indication for TSB**	2	44	Identified by visual inspection: 46
	Missed by TcB: 2	Identified by TcB: 72	

Abbreviations: TcB, transcutaneous bilirubin; TSB, total serum bilirubin

Number of neonates with TSB level below the treatment threshold requiring unnecessary blood pricks by TcB versus visual inspection

	TcB reading below threshold	TcB reading above threshold
**No visual indication for TSB**	0	222
**Visual indication for TSB**	62	87

Abbreviations: TcB, transcutaneous bilirubin; TSB, total serum bilirubin

© 2026 Westenberg LH et al. *JAMA Network Open*.

eAppendix 11. Corresponding 2 × 2 Tables Sensitivity Analysis Excluded Neonates Assessed in a Particular Hospital-Laboratory

11A – Sensitivity analysis excluding one hospital-laboratory

Number of neonates with TSB level above the treatment threshold identified by TcB versus visual inspection

	TcB reading below threshold	TcB reading above threshold	
No visual indication for TSB	0	10	Missed by visual inspection: 10
Visual indication for TSB	3	15	Identified by visual inspection: 18
	Missed by TcB: 3	Identified by TcB: 25	

Abbreviations: TcB, transcutaneous bilirubin; TSB, total serum bilirubin

11B – Sensitivity analysis excluding one hospital-laboratory and per-protocol analysis

Number of neonates with TSB level above the treatment threshold identified by TcB versus visual inspection

	TcB reading below threshold	TcB reading above threshold	
No visual indication for TSB	0	10	Missed by visual inspection: 10
Visual indication for TSB	0	18	Identified by visual inspection: 18
	Missed by TcB: 0	Identified by TcB: 28	

Abbreviations: TcB, transcutaneous bilirubin; TSB, total serum bilirubin

© 2026 Westenberg LH et al. *JAMA Network Open*.

eAppendix 12. Unit Prices Used in the Cost-Effectiveness Analysis

Health care resource	Price in Euros
Transcutaneous bilirubin meter, per measurement (including purchase, maintenance and calibration)	€ 1.94
Phototherapy treatment in hospital, per 24 hours (including purchase, maintenance, calibration, and goggles)	€ 21.13
Intensive phototherapy treatment in hospital, per 24 hours (including purchase, maintenance, calibration, and goggles)	€ 42.26
Phototherapy treatment at home, per 24 hours (including purchase, maintenance, calibration, goggles, time spent by midwives on instructing parents, and consultations with a pediatrician)	€ 68.71
Total serum bilirubin quantification in laboratory	€ 6.85
Transportation of blood samples to local laboratory (including 30 minutes health care personnel)	€ 34.18
(Telephone) consultation midwife, per 15 minutes	€ 17.09
(Telephone) consultation with pediatrician, per 15 minutes	€ 38.48
Inpatient hospital day on the ward	€ 760
Inpatient hospital day on the ICU	€ 3,216
Blood products ordered for exchange transfusion (without the neonate actually receiving the treatment)	€ 1,617
Exchange transfusion treatment (including five ICU hospital days, blood products, and health care personnel)	€ 21,486

To determine costs, resource use was multiplied by integral cost prices, which included both personnel and material costs. These unit costs were based on Dutch reference prices and on data received from equipment manufacturers. All costs were calculated in Euros and expressed in 2025 price levels.

Abbreviations: ICU, intensive care unit

* The calculations assumed that the bilirubin meter had a lifespan of 7 years and that a midwife practice used the device 2-3 times per day.

© 2026 Westenberg LH et al. *JAMA Network Open*.
