# Supplementary material for: Universal Transcutaneous Bilirubin Screening in a Midwifery-Led Home Care Setting
Source: JAMA Netw Open. 2026 Jan 12;9(1):e2551883. doi: 10.1001/jamanetworkopen.2025.51883 (PMC12797098; doi:10.1001/jamanetworkopen.2025.51883)
Supplement: Supplement 2. — Nonauthor Collaborators [file jamanetwopen-e2551883-s002.pdf]

Supplemental Online Content: Nonauthor Collaborators
*First name, last name, and suffix (if applicable) are required and will appear in PubMed.

*Group Name(s): BEAT study group							
***First Name and Middle Initial(s)**	***Last Name**	***Suffix (eg, Jr, III)**	Academic Degrees	Institution	Location (city, state/province, country)	Role or Contribution, eg, chair, principal investigator	Group (if more than 1 Group listed in the byline) and/or Subgroup (eg, Steering Committee)
Hanneke	Bontenbal-Pleizier		NA	Midwifery practice Charlois	Rotterdam, the Netherlands	Study group member	BEAT study group
Jan Jaap	Erwich		Prof	Department of Obstetrics and Gynecology, Amsterdam Public Health Research Institute, VU University Medical Center	Groningen, The Netherlands	Study group member	BEAT study group
Esther I.	Feijen-de Jong		NA	Department of Midwifery Science, Amsterdam Public Health Research Institute, VU University Medical Center	Amsterdam, The Netherlands	Study group member	BEAT study group
Hanneke W.	Harmsen van der Vliet-Torij		Doctorate	Research Center Innovations in Care, Rotterdam University of Applied Sciences,	Rotterdam, the Netherlands	Co-applicant funding, Study group member	BEAT study group
Eline	van Waes-Hemminga		NA	Midwifery practice Rotterdam Oost	Rotterdam, the Netherlands	Study group member	BEAT study group
Femke	Karels		NA	Midwifery practice Bergweg	Rotterdam, the Netherlands	Study group member	BEAT study group
Dorien	Kraima		NA	Midwifery practice Veendam	Veendam, the Netherlands	Study group member	BEAT study group
Jenneke	Kruidhof		NA	Midwifery practice Fiere	Groningen, the Netherlands	Study group member	BEAT study group
Celine	Leonard		NA	Midwifery practice Lansingerland	Bergschenhoek, The Netherlands	Study group member	BEAT study group
Gea	Reussing		NA	Midwifery practice NOP/Lemsterland	Emmeloord, The Netherlands	Study group member	BEAT study group
Joyce	Vaandrager		NA	Midwifery practice IJsselmonde	Rotterdam, the Netherlands	Study group member	BEAT study group
Ellen	Vreugdenhil		NA	Midwifery practice Midden Groningen	Hoogezand, The Netherlands	Study group member	BEAT study group
A. H.	Westenberg		MD	Erasmus Medical Center Rotterdam	Rotterdam, the Netherlands	Study group member	BEAT study group
