# Supplementary material for: Universal Transcutaneous Bilirubin Screening in a Midwifery-Led Home Care Setting
Source: JAMA Netw Open. 2026 Jan 12;9(1):e2551883. doi: 10.1001/jamanetworkopen.2025.51883 (PMC12797098; doi:10.1001/jamanetworkopen.2025.51883)
Supplement: Supplement 3. — Data Sharing Statement [file jamanetwopen-e2551883-s003.pdf]

Data Sharing Statement

Westenberg. Universal Transcutaneous Bilirubin Screening in a Midwifery-Led Home Care Setting. *JAMA Netw Open*. Published January 09, 2026.
doi:10.1001/jamanetworkopen.2025.51883

Data
Data available: No

Additional Information
Explanation for why data not available: Metadata will be made available in Dataverse NL, which we are currently in the process of organizing. The data can be accessed under restricted conditions. If data is shared, this is done through a data transfer agreement.
